# The effects of myocardial bridging on two-dimensional myocardial strain during dobutamine stress echocardiography

**DOI:** 10.1007/s10554-024-03239-z

**Published:** 2024-09-13

**Authors:** Haitham Ballo, Valtteri Uusitalo, Mikko Pietilä, Maria Wendelin-Saarenhovi, Markku Saraste, Juhani Knuuti, Antti Saraste

**Affiliations:** 1 Turku PET Centre, Turku University Hospital and University of Turku, Turku, Finland; 2 Heart Center, Turku University Hospital and University of Turku, Hämeentie 11, 20520 Turku, Finland; 3 Clinical Physiology and Nuclear Medicine, Helsinki University Hospital and University of Helsinki, Helsinki, Finland; 4 Clinical Physiology, Nuclear Medicine and PET, Turku University Hospital and University of Turku, Turku, Finland

**Keywords:** Myocardial bridge, Myocardial strain, Dobutamine stress echocardiography

## Abstract

Myocardial bridging (MB) is a common anatomic variant in coronary arteries with unclear functional significance. We evaluated regional myocardial strain by speckle tracking during dobutamine stress echocardiography (DSE) in patients with MB in the left anterior descending coronary artery (LAD). We studied 11 patients with MB in the LAD and no obstructive coronary artery disease (CAD), 7 patients without MB, but obstructive CAD in the LAD, and 12 controls without MB or obstructive CAD. MB was defined as either > 1 mm (superficial) or > 2 mm (deep) intramyocardial course of the LAD in coronary CT angiography. Regional longitudinal, radial and circumferential strains and strain rates as well as post-systolic strain index (PSI) were measured at rest, peak stress, and early recovery (1 min after stress). Strain parameters during DSE were similar in the myocardium distal to MB and other myocardial regions of the same patients as well as the LAD territory in controls. However, patients with obstructive CAD showed impaired LS and strain rate as well as increased PSI at peak stress. None of the MB was associated with systolic compression in invasive coronary angiography and strain parameters were similar between superficial and deep MB. Stress myocardial blood flow by positron emission tomography correlated with LS and RS at peak stress in the myocardium distal to MB (r = − 0.73, p = 0.03, and r = 0.64, p = 0.04, respectively). Myocardial strain is not reduced during DSE in patients with MB in the LAD and no significant systolic compression.

## Introduction

Myocardial bridging (MB) is a common anatomical variant characterized by a segment of a coronary artery covered by a myocardial band, also referred to as a tunneled artery [1]. The incidence of MB is highest in the left anterior descending coronary artery (LAD) [2]. Although the long-term prognosis of patients with MB is good [3, 4], some studies reported that MB could be associated with anginal pain, impaired systolic function, and even cardiac death [5–7].

The presence of a MB can be seen as systolic compression of a coronary artery segment by invasive coronary angiography (ICA). More frequently, MB is detected by coronary computed tomography angiography (CTA) as an intramyocardial course of the coronary artery [8, 9]. However, anatomical imaging alone does not provide information on the hemodynamic significance of the myocardial bridging and its significance to the patients’ symptoms which is crucial in guiding further clinical management. Studies using positron emission tomography (PET) have found normal myocardial blood flow (MBF) during vasodilator stress in myocardium subtended by MB [10, 11]. However, the vasodilator stress might not be optimal for the evaluation of dynamic compression caused by the MB compared to exercise or dobutamine stress [12].

Dobutamine stress echocardiography (DSE) detects obstructive coronary artery disease (CAD) based on regional wall motion abnormalities induced by myocardial ischemia [13, 14]. Two-dimensional strain echocardiography (speckle-tracking echocardiography, STE) detects early signs of left ventricular dysfunction in patients with CAD [15]. Furthermore, STE during DSE was recently found feasible and may help to detect hemodynamically significant coronary stenosis compared with visual analysis alone [16, 17]. While wall motion abnormalities associated with MB have been found in some patients during stress echocardiography [18], there has been an interest in the use of strain imaging to evaluate the hemodynamic effects of MB [19].

In this study, we evaluated the relationships between the regional two-dimensional myocardial strain measured by STE during DSE and the anatomical and functional characteristics of MB in the LAD.

## Methods

The study group consisted of 50 prospectively recruited outpatients evaluated during years 2009–2011 due to suspected obstructive CAD with an intermediate pre-test likelihood of CAD that has been described previously [16]. A subpopulation of a study evaluating diagnostic approach using hybrid coronary CTA/PET perfusion imaging for diagnosis of obstructive CAD defined by invasive coronary angiography as the reference standard underwent additional DSE [20]. Exclusion criteria were age < 30 or > 75 years, low or high pre-test probability of CAD, pregnancy, acute coronary syndrome, known diagnosis of CAD (history of myocardial infarction or obstructive CAD by ICA), ejection fraction < 35%, asthma, significant valvular disease, congenital heart disease, cardiomyopathy, severe hypertension, recent (< 6 months) cerebral ischemic attack, active cancer, persistent atrial fibrillation, and atrioventricular block. The study was performed according to the Declaration of Helsinki, it was approved by the local ethics committee and participants gave their informed written consent.

According to the study protocol, all patients underwent DSE, coronary CTA, PET perfusion imaging during adenosine stress, and invasive coronary angiography, which was performed on average 5 ± 3 weeks after DSE.

For this analysis, we included 11 patients with MB in the LAD without obstructive CAD, 7 patients with obstructive CAD in the LAD, and 12 control patients without MB or obstructive CAD. Six patients with concomitant MB and obstructive CAD in the LAD or diagonal branches were excluded.

### Dobutamine stress echocardiography

A standard staged protocol was applied during DSE [13, 14]. Dobutamine was infused intravenously through a peripheral infusion line with a mechanical pump starting at a dose of 10 µg/kg/min. The dose was increased at 3-min intervals to 20, 30, and 40 µg/kg/min with intravenous atropine up to 2 mg given if necessary to augment the heart rate response. Blood pressure and electrocardiogram were monitored continuously. Criteria for terminating the test were achieving a heart rate response of 85% of the age-predicted maximum, development of wall motion abnormality, angina pectoris, severe ischemic electrocardiographic changes, systolic blood pressure > 240 mmHg, abnormal blood pressure reaction during stress, or significant arrhythmia. Beta-blockers were withdrawn for two days and long-acting nitrates on the morning of the study.

### Image acquisition

Images were obtained by three experienced echocardiographers using the GE Vivid 7 and M4S transducer (GE Vingmed Ultrasound AS, Horten, Norway) with patients in the lateral decubitus position. Standard 2D grayscale images of three standard apical views (four-chamber, two-chamber, and apical long-axis) and parasternal long-axis and parasternal short-axis views at the level of the mitral valve, papillary muscles, and apex were acquired at rest, at a dobutamine dose of 20 mg/kg/min, at peak stress, and early recovery 1 min after stress. Per protocol, a cine image of one representative cardiac cycle per stage and view was digitally stored for later offline analysis. To enhance speckle-tracking at high heart rates, images were optimized for left ventricular analysis, and the frame rate was increased to achieve a target of 60 to 90 frames/sec without compromising endocardial border detection.

### Two-dimensional speckle-tracking strain analysis

The speckle-tracking strain was measured offline using EchoPAC PC 113 software (GE Healthcare Inc.) by an analyst blinded to the other imaging results of the patient. Semi-automatic software created a region of interest (ROI) covering the LV myocardium. Speckles were assumed automatically by the software, and the user confirmed proper tracking of the myocardium. In the presence of poor tracking, the observer re-adjusted the endocardial trace. Segments rejected both by software and by the analyst were excluded. In patients with MB, 4 segments in the LAD territory with poor tracking were excluded at rest, 3 at peak stress and 2 at early recovery. In the control group, 5 segments were excluded at rest, 5 at peak stress, and 6 at early recovery.

Regional systolic longitudinal strain (LS), longitudinal strain rate (LSr), circumferential strain (CS), circumferential strain rate (CSr), radial strain (RS) and radial strain rate (RSr) were calculated in all LV segments. Furthermore, we measured postsystolic strain index (PSI, postsystolic strain divided by peak strain). Strain values were calculated for 18 myocardial segments using the standard template. In the presence of obstructive stenosis or MB, distal myocardial segments were chosen for strain analysis. In patients with MB, segments outside the LAD territory were analyzed as control segments.

### Coronary computed tomography angiography

Patients were scanned with a 64-row PET/CT scanner (GE Discovery VCT, General Electric Medical Systems, Waukesha, Wisconsin) as previously described [11, 16]. The collimation was 64 × 0.625 mm, gantry rotation time was 350 ms, tube current was 600 to 750 mA, and voltage was 100 to 120 kV, depending on patient size. Patients received 800 μg sublingual nitrate before the scan. Intravenous metoprolol 0 to 30 mg was administered before the scan to reach a target heart rate of 60 bpm. Iodinated contrast infusion (60 to 80 ml of 400 mg iodine/ml iomeprol at 4 to 4.5 ml/s) was followed by a saline flush.

Coronary CTA was analysed according to the standard 17-vessel segment model [21]. To evaluate the presence of MB, multiplanar reconstruction images were used. MB was defined as an intramyocardial course of a coronary artery, either superficial (1 to 2 mm) or deep (> 2 mm of overlying myocardium) according to the definitions used in the previous studies [4, 8, 11, 22, 23].

### Positron emission tomography

PET studies were performed using Discovery VCT scanner (GE Medical Systems, Waukesha, WI), as previously described [11, 16] after an overnight fast. Alcohol and caffeine were prohibited 12 h before the study. Rest-stress perfusion cardiac PET was performed immediately after CT, and 15O-labeled water (900 to 1100 MBq) was injected (RadiowaterGenerator, Hidex Oy, Turku, Finland) at rest as an intravenous bolus over 15 s. A dynamic acquisition of the heart was performed (14 × 5 s, 3 × 10 s, 3 × 20 s, and 4 × 30 s), after which an adenosine-induced stress scan was performed. Adenosine infusion was started 2 min before the scan and continued at 140 mg/kg/min until the scan was completed.

**Fig. 1:**
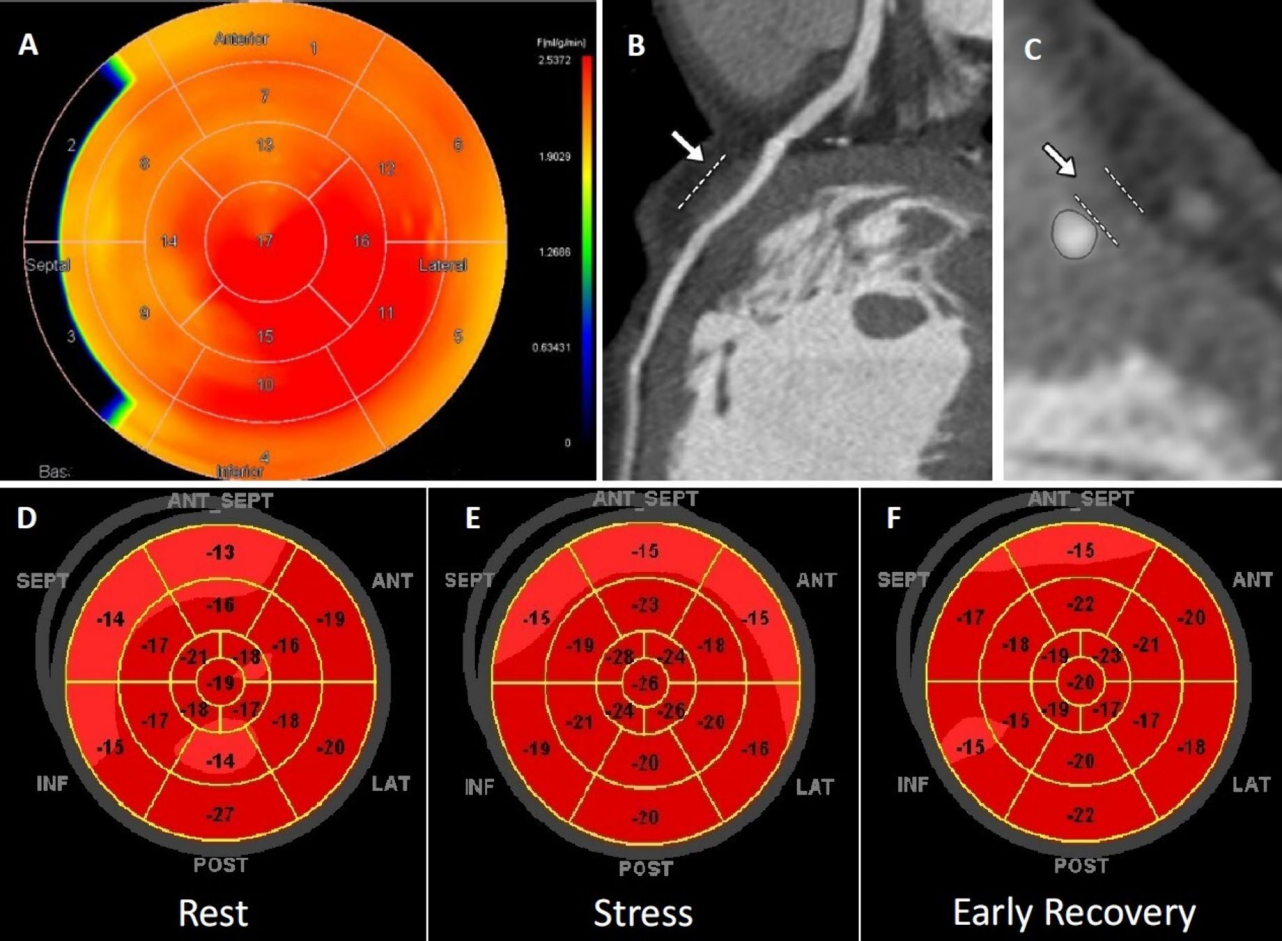
Positron emission tomography (PET), computed tomography angiography (CTA), and dobutamine stress echocardiography (DSE) of a patient with myocardial bridging (MB) in the middle left anterior descending (LAD) coronary artery. A polar map of stress myocardial perfusion measured using 15-Oxygen water PET shows normal stress myocardial blood flow (**A**). Multiplanar reconstruction (**B**) and cross-sectional (**C**) images of a MB in the mid-LAD artery. White arrows and lines demonstrate the myocardium overlying the artery. Representative polar maps of peak systolic longitudinal strain at the rest (**D**) peak dobutamine stress (**E**) and early recovery (**F**) phases. Myocardial strain is homogeneous throughout the test

PET images were analyzed with Carimas software [24] and MBF was expressed as ml/g/min. Standard polar plots and a parametric heart volume were produced, allowing image fusion with ADW 4.4 software (CardiIQFusion, General Electric Medical Systems, Milwaukee, Wisconsin). PET/CT hybrid images were used to match coronary artery segments affected by MB to corresponding myocardial flow areas. Myocardial segments for flow analysis were chosen distal to MB. An example of PET perfusion imaging of MB is shown in Fig. 1.

### Invasive coronary angiography and FFR

ICA was performed with Siemens Axiom Artis coronary angiography system (Siemens, Erlangen, Germany) as previously described15. An experienced reader blinded to the results of the other imaging modalities performed quantitative analysis of coronary angiograms (Quantcore, Siemens) for CAD and assessment for systolic compression. A 17-segment standard model was used. FFR measurement was performed using the ComboMap pressure/flow instrument and 0.014-inch Bright Wire pressure guidewires (Volcano Corporation, San Diego, CA) in the presence of intermediate stenosis of 40% to 75% when feasible. The pressure was measured distally to the lesion during maximal hyperaemia induced by 18-mg intracoronary boluses of adenosine with simultaneous measurement of aortic pressure through the catheter. FFR was calculated as the ratio of mean distal pressure to mean aortic pressure.

Obstructive CAD was defined as > 70% anatomic stenosis by quantitative coronary angiography or in the presence of an intermediate stenosis (40–75%) as FFR < 0.8. In one patient without FFR measurement, the presence of myocardial ischemia detected by PET perfusion imaging confirmed CAD.

### Statistical analysis

A Shapiro–Wilk W-test was applied to test the distribution of the data. All variables were expressed as the mean ± standard deviation (SD). Chi-square tests were used for differences between categorical variables and Student's t-tests for continuous variables. Analysis of variance (ANOVA) and Tukey Kramer tests were used to assess differences among three groups. The Pearson correlation coefficient between myocardial strain values and MBF was calculated. A two-sided p-value < 0.05 was considered to indicate significance. Statistical analyses were done using SPSS (SPSS, Chicago, IL).

## Results

The study group consisted of 11 patients with MB in the LAD and no obstructive CAD (one patient had an intermediate stenosis, but fractional flow reserve was normal), 7 patients with obstructive CAD in the LAD, and 12 controls without MB or obstructive CAD. The MB was in the middle part of the LAD in 9 patients and distal part in 2 patients. Six patients had a deep course of the MB segment. The mean MB length was 13.2 ± 4.7 mm, and the longest MB was 21 mm. None of the MB was associated with > 50% systolic compression by ICA.

Table 1 shows the clinical characteristics of study patients. The proportion of women, age, type of symptoms, prevalence of risk factors of CAD and medications were similar among patients with MB, obstructive CAD and controls. Systemic hemodynamic response and heart rate response to dobutamine stress were similar among patients with MB, CAD and controls (Table 2).

**Table 1:** Clinical characteristics of study patients

	All (N = 30)	MB (N = 11)	Control (N = 12)	CAD (N = 7)	*p* value
Men	17 (57)	7 (64)	7 (58)	3 (43)	0.8
Age, years (mean ± SD)	63 ± 7	61 ± 9	63 ± 5	65 ± 7	0.6
Symptom*					
Typical angina	7 (23)	2 (18)	3 (25)	2 (29)	0.7
Atypical angina	14 (47)	5 (45)	5 (41)	4 (57)	0.6
Non-anginal chest pain	8 (27)	4 (36)	3 (25)	1 (14)	0.7
*Risk factors*					
Hypertension	11 (37)	4 (36)	4 (33)	3 (43)	0.8
Hypercholesterolemia	14 (47)	6 (55)	5 (41)	3 (43)	0.7
Diabetes	4 (13)	0	2 (17)	2 (29)	0.2
Current or previous smoker	4 (13)	1(9)	1 (8)	2 (29)	0.2
*Medication*					
Aspirin	16 (53)	5 (45)	6 (50)	5 (71)	0.5
Beta blocker	17 (57)	7 (64)	5 (42)	5 (71)	0.5
Statin	17 (57)	8 (73)	5 (42)	4 (57)	0.3
ACE inhibitor/ATr-antagonist	6 (20)	1 (9)	2 (17)	3 (43)	0.2
Calcium channel blocker	3 (10)	0	2 (17)	1 (14)	0.4

Data are presented as n (%) unless otherwise stated

*MB* Myocardial bridging, *CAD* coronary artery disease, *ACE* angiotensin-converting enzyme, *ATr* angiotensin 2 receptor, *FFR* fractional flow reserve, *PET* positron emission tomography, *SD* standard deviation

*In one patient information on symptoms missing

**Table 2:** Systemic hemodynamic variables and left ventricular ejection fraction (LVEF) at rest and peak dobutamine stress

Variables	MB (N = 11)	Control (N = 12)	CAD (N = 7)	*p* value (ANOVA)
*Systolic blood pressure (mmHg)*				
Rest	131 ± 14	136 ± 17	147 ± 21	0.19
Peak stress	151 ± 32	148 ± 18	157 ± 22	0.75
*Heart rate (beats/min)*				
Rest	65 ± 6	70 ± 12	67 ± 11	0.46
Peak stress	147 ± 24	143 ± 17	151.2 ± 35	0.76
*Rate pressure product (mmHg/min)*				
Rest	8460 ± 1181	9815 ± 2447	10,356 ± 2893	0.19
Maximum	21,054 ± 7001	19,093 ± 3324	24,401 ± 7619	0.17
*LVEF (%)*				
Rest	68 ± 5	67 ± 5	70 ± 3	0.39
Peak stress	79 ± 7	77 ± 8	77 ± 9	0.89

Data are presented as mean ± SD

*MB* Myocardial bridging, *CAD* coronary artery disease

Standard echocardiography showed normal left ventricular ejection fraction, no scars of myocardial infarction nor other significant structural heart disease (Table 2). At rest, the visual wall motion in the LAD territory was normal in all patients except one patient with obstructive CAD who showed hypokinesia in one segment. At peak stress, inducible wall motion abnormalities were seen in the LAD territory in 3 patients with an obstructive CAD (1, 3 and 7 segments per patient), whereas no wall motion abnormality was seen in patients with MB or controls.

### Two-dimensional strain during dobutamine stress in patients with MB

An example of longitudinal strain maps during DSE in a patient with MB is shown in Fig. 1. Assessment of 2D strain was feasible during DSE in all patients. As shown in Table 3, all strain parameters were similar in the myocardium distal to coronary segments with MB and remote-control segments of the same patients at rest, peak dobutamine stress, and early recovery. Compared with rest, LS, LSr, CSr, RS and RSr were augmented at peak dobutamine stress in segments distal to MB. However, there were no significant changes in PSI and CS during dobutamine stress. At all-time points of DSE, strain parameters distal to MB with superficial vs. deep intramyocardial course were similar (Table 4).

**Table 3:** Myocardial strain distal to coronary artery segments with myocardial bridging (MB) versus remote myocardial region of the same patients at rest and dobutamine stress

Strain	Rest			Peak stress			Early recovery		
	MB	Remote	*p* value	MB	Remote	*p* value	MB	Remote	*p* value
LS (%)	− 19.1 ± 2.6*	− 17.7 ± 1.6	0.18	− 23.4 ± 6.3	− 19.2 ± 2	0.07	− 21.3 ± 3.8	− 18.8 ± 2.2	0.06
LSr (S^−1^)	− 1 ± 0.2*	− 1 ± 0.1*	0.78	− 2 ± 0.9	− 2.4 ± 0.2**	0.07	− 2.1 ± 0.8	− 1.9 ± 0.3***	0.20
PSI (%)	1.3 ± 1.7	1.7 ± 1	0.5	2.7 ± 1.8	3.4 ± 1.4	0.30	2.3 ± 2.1	3.1 ± 1.9	0.3
CS (%)	− 20.3 ± 4.8	− 16.9 ± 2.5	0.17	− 24.2 ± 7.9	− 19.9 ± 3.4	0.14	− 25.3 ± 7.6	− 20.3 ± 5.2	0.12
CSr (S^−1^)	− 1.8 ± 0.5*	− 1.9 ± 0.2*	0.65	− 4.2 ± 1.1	− 3.8 ± 0.6	0.21	− 3.5 ± 1.3	− 3.2 ± 0.9	0.63
RS (%)	32.1 ± 11.8*	33 ± 6.5	0.86	45.6 ± 13.9	38.8 ± 8.4	0.21	41.5 ± 26.1	37.3 ± 21.5	0.71
RSr (S^−1^)	2.4 ± 0.5*	2.4 ± 0.3*	0.93	7.2 ± 2.5	6.1 ± 1.9	0.32	5.8 ± 2.1	6.1 ± 1.8***	0.76

Data are presented as mean ± SD

*LS* Longitudinal strain, *LSr* Longitudinal strain rate, *PSI* Post systolic index, *CS* Circumferential strain, *CSr* Circumferential strain rate, *RS* Radial strain, *RSr* Radial strain rate

*vs. stress*, P* < 0.05. **vs. recovery,* P* < 0.05. ***vs. rest,* P* < 0.05

**Table 4:** Myocardial regional strain distal to coronary artery segments with superficial versus deep myocardial bridging (MB) at rest and dobutamine stress

Strain	Rest			Peak stress			Early recovery		
	Superficial	Deep	*p* value	Superficial	Deep	*p* value	Superficial	Deep	*p* value
LS (%)	− 20.2 ± 2.9	− 18.6 ± 2.2	0.40	− 23.9 ± 6.3	− 23.3 ± 11.1	0.85	− 21.7 ± 4.8	− 20.9 ± 9.7	0.8
LSr (S^−1^)	− 1.1 ± 0.2	− 1 ± 0.5	0.40	− 3.1 ± 1	− 2.9 ± 1.6	0.70	− 2.1 ± 0.4	− 2 ± 1.1	0.4
PSI (%)	0.8 ± 0.9	1.8 ± 2.2	0.4	2.8 ± 1.5	2.6 ± 2.3	0.9	1.2 ± 0.9	2.8 ± 2.4	0.2
CS (%)	− 18.8 ± 3.9	− 21.6 ± 5.7	0.40	− 24.7 ± 9.5	− 23.1 ± 2.4	0.70	− 22.9 ± 7.9	− 27.6 ± 4.1	0.1
CSr (S^−1^)	− 2.1 ± 0.9*	− 1.8 ± 0.3*	0.4	− 4.9 ± 1	− 3.7 ± 0.5	0.6	− 3.8 ± 0.7**	− 3.6 ± 1.2**	0.8
RS (%)	29.6 ± 10	32.4 ± 11	0.09	35.2 ± 20	40.9 ± 18	0.49	31.70 ± 20	39.8 ± 12.5	0.6
RSr (S^−1^)	2.90 ± 1.3*	2.7 ± 0.6*	0.80	9.70 ± 4.7	6.60 ± 1.9	0.60	5.5 ± 1.7**	6.9 ± 2.2**	0.3

Data are presented as mean ± SD

*LS* Longitudinal strain, *LSr* Longitudinal strain rate, *PSI* Post systolic index, *CS* Circumferential strain, *CSr* Circumferential strain rate, *RS* Radial strain, *RSr* Radial strain rate

*vs. stress*, P* < 0.05. **vs. rest,* P* < 0.05

### Two-dimensional strain during dobutamine stress in patients with MB, obstructive CAD and controls

**Fig. 2:**
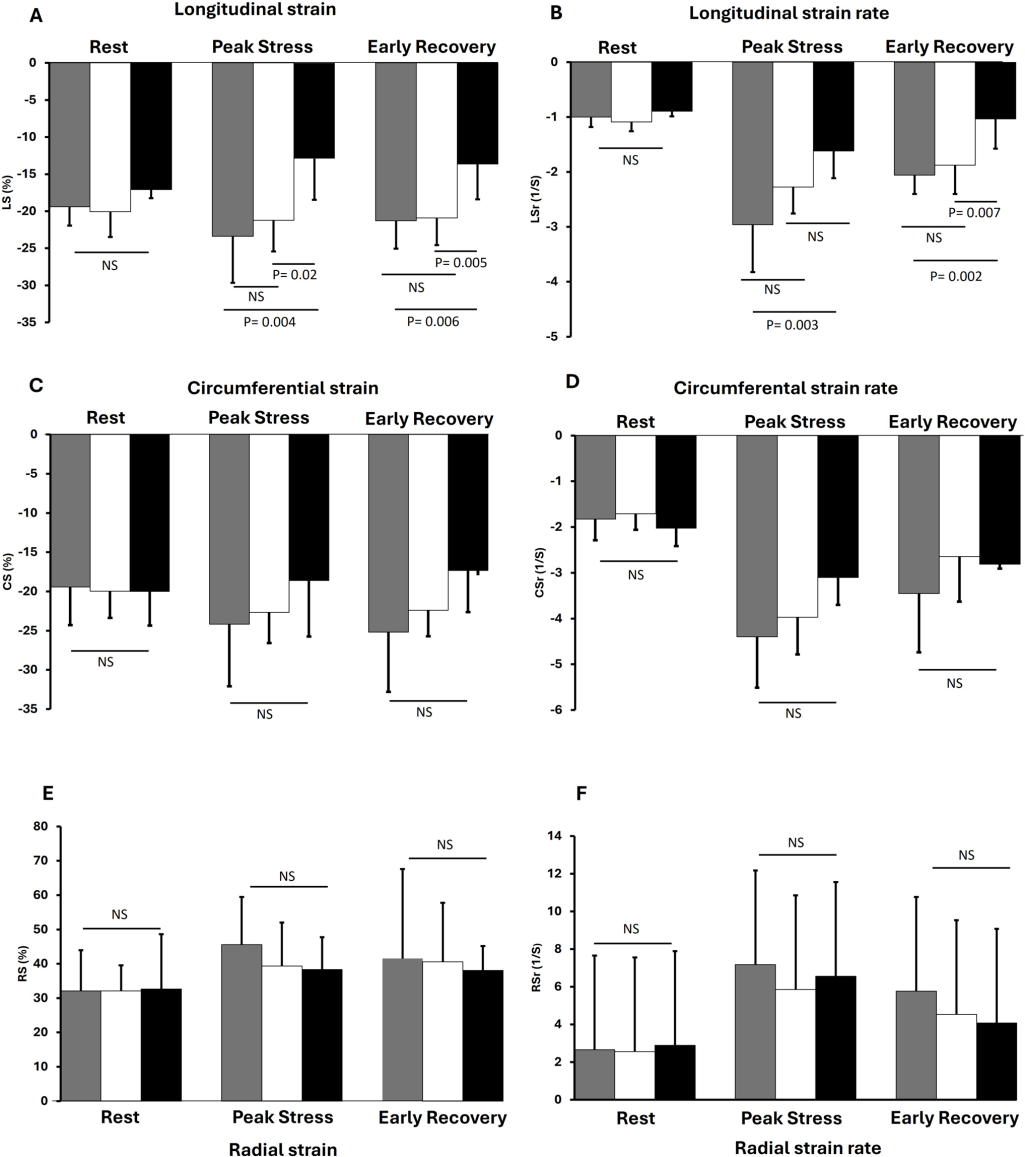
Longitudinal strain (**A**), longitudinal strain rate (**B**), circumferential strain (**C**), circumferential strain rate (**D**), radial strain (**E**), and radial strain rate (**F**) distal to MB (Gray), distal obstructive CAD lesions (black) and in the myocardial region subtended by the LAD in controls (white) in different phases of dobutamine stress echocardiography. *NS* non-significant

**Fig. 3:**
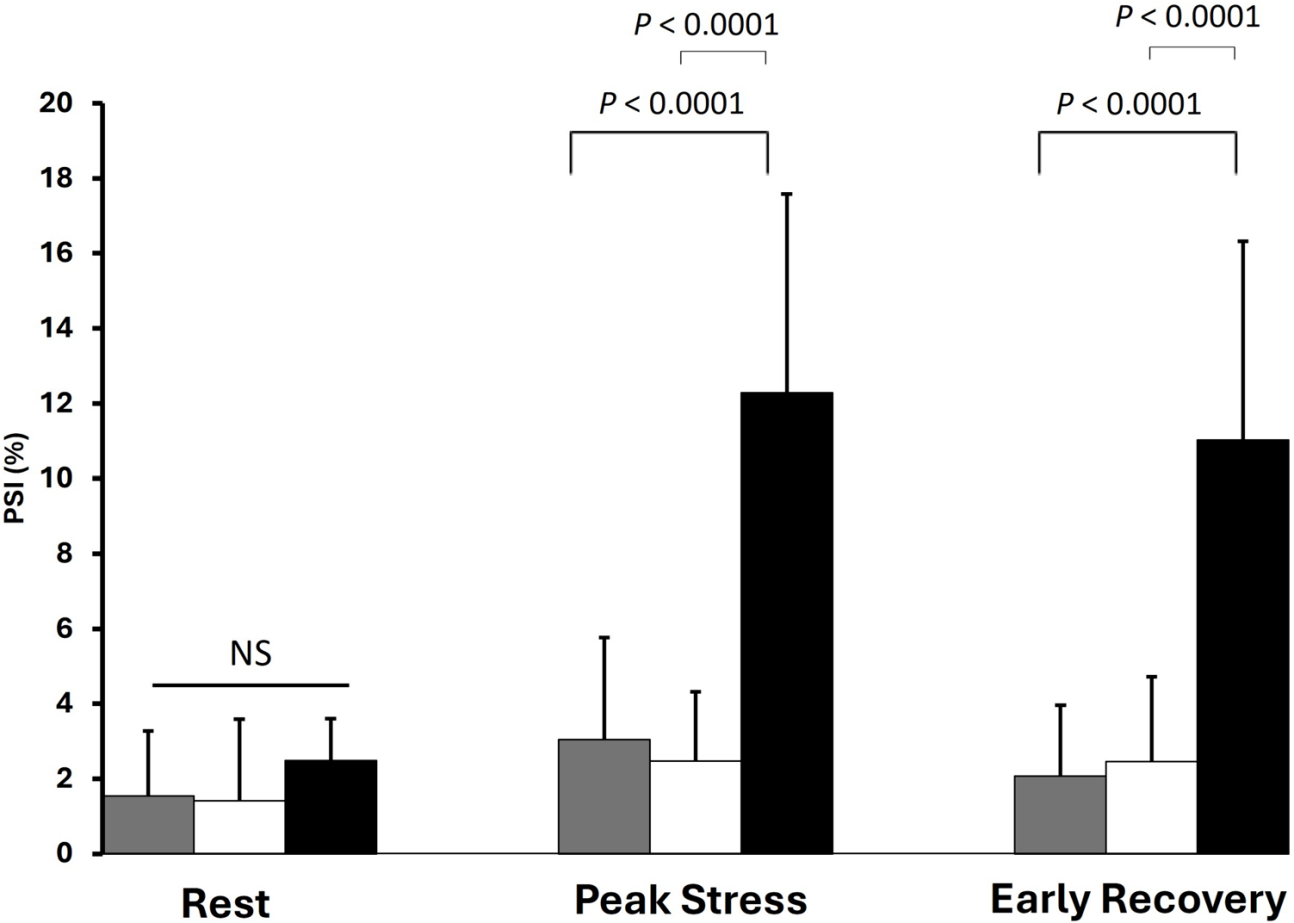
Regional post-systolic strain index (PSI) distal to MB (Gray), distal to obstructive CAD lesion (black) and corresponding myocardium of controls (white) at different phases of dobutamine stress echocardiography. *NS* non-significant

Myocardial strain parameters at rest were similar distal to MB, distal to obstructive CAD lesions and in the corresponding myocardium subtended by the LAD in controls as shown in Fig. 2. During peak stress and early recovery, LS was comparable between MB and control groups (− 23.4 ± 6.3% vs. − 21.3 ± 4.4%, p > 0.05, and − 21.3 ± 3.8% vs. − 21.19 ± 3.7%, p > 0.05, respectively). However, LS in the CAD group was reduced as compared to MB and control groups during peak stress and early recovery (− 12.9 ± 5.6%, p < 0.05 for both groups and − 13.63 ± 4.7%, p < 0.05 for both groups, respectively). Similarly, LSr was comparable between MB and control groups during peak stress and early recovery phases (− 2.0 ± 0.9 S^−1^ vs. − 2.1 ± 2 S^−1^, p > 0.05, and − 2.1 ± 0.8 vs. − 1.9 ± 0.3 S^−1^ p > 0.05, respectively). LSr was lower in the CAD group than MB group during peak stress (− 1.6 ± 0.5 S^−1^, p = 0.003) and lower than MB and control groups during early recovery (− 1.0 ± 0.5 S^−1^, p = 0.002, and − 1.0 ± 0.5 S^−1^, p = 0.007, respectively). Furthermore, regional PSI was significantly higher in CAD than in MB and control groups both at peak stress (All p < 0.0001) and early recovery (All p < 0.0001) as shown in Fig. 3. CS, CSr, RS and RSr showed no significant differences between MB, CAD and control groups during dobutamine stress.

### Relation between stress myocardial blood flow and two-dimensional strain during dobutamine stress in patients with MB

**Fig. 4:**
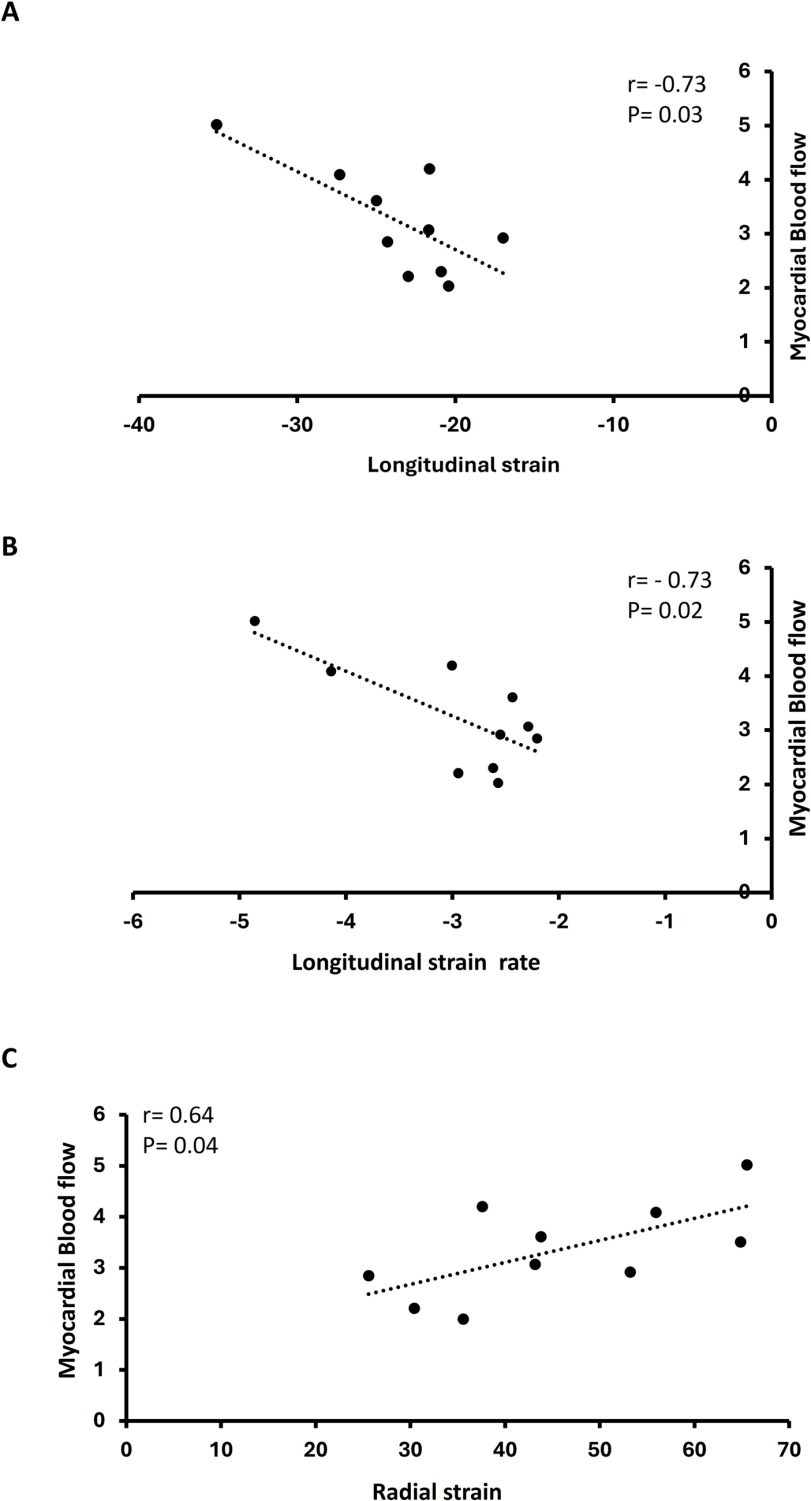
Correlations between myocardial two-dimensional strain at peak dobutamine stress and myocardial blood flow during adenosine stress distal to myocardial bridging. Pearson correlation (r-value) and probability (p-value) are indicated for longitudinal strain (**A**), longitudinal strain rate (**B**), and Radial strain (**C**)

Regional MBF during adenosine stress correlated with regional LS (r = − 0.73, p = 0.03), LSr (r = − 0.73, p = 0.02) and RS (r = 0.64, p = 0.04) at peak stress in the myocardium subtended by MB (Fig. 4). There were no correlations between stress MBF and other strain variables at dobutamine stress. Similarly, resting MBF and myocardial strain parameters at rest were not correlated.

## Discussion

In the present study, we studied the effect of MB on two-dimensional myocardial strain measured by speckle tracking during DSE. The main finding of the study is that myocardial strain parameters were similar distal to the MB vs. myocardium subtended by a normal coronary artery of the same patients or the corresponding myocardial region of control patients without CAD or MB during dobutamine stress. In contrast to MB, obstructive CAD was associated with reduced LS and LSr as well as increased PSI during DSE. Although our study population is not representative of different anatomical variants of MB, our results provide evidence that the presence of MB seen as intramyocardial course of the LAD by coronary CTA without systolic compression in invasive coronary angiography, is not typically associated with altered myocardial strain during high dose dobutamine stress.

Evaluation of the hemodynamic consequences of MB remains a diagnostic challenge. Previous studies using positron emission tomography indicate that MB does not affect absolute myocardial blood flow in response to vasodilator stress [10, 11]. However, another recent study found that MB in the LAD was associated with reduced coronary flow velocity reserve as well as increased frequency of reversible myocardial perfusion defects and wall motion abnormalities in response to vasodilator stress [18].

Increased heart rate in response to exercise or dobutamine stress changes the relative length of systole to diastole and increases myocardial contractility which might be more effective in uncovering possible physiologic effects caused by MB than vasodilator stress. Kobayashi et al. [25] found reduced 2D myocardial strain in the interventricular septum during exercise stress in 55 patients with MB detected by intravascular ultrasound in the LAD. The reduction was associated with decreases in diastolic fractional flow reserve distal to MB in response to dobutamine. Using 2D STE, Jhi et al. [26]. found limited increase in longitudinal, radial and circumferential strain in the anterior and septal myocardial segments during low dose (20 μg/kg/min) DSE in 18 patients with MB in the LAD causing systolic compression. During high-dose DSE, ischemic wall motion abnormalities were found in 14% of patients with MB in the LAD and cyclic variation of integrated backscatter was significantly reduced [27]. More recently, studies have indicated that measurement of systolic strain by STE during high-dose DSE may be feasible and may help in detection of hemodynamically significant CAD and subtle LV segmental dysfunction [16, 17]. In our study, regional strain parameters, including PSI, distal to MB in the LAD were comparable to strain measurements in the remote myocardium not affected by MB or CAD of the same patient during all phases of DSE. Moreover, the regional strain measurements distal to MB were similar to strain in the corresponding LAD territory of patients with neither MB nor CAD. This was in contrast to patients with obstructive CAD in whom LS and LSr were impaired, and PSI increased during dobutamine stress.

In our study, none of the myocardial bridges was associated with > 50% systolic compression in ICA. Wang et al. [19]. evaluated the influence of isolated MB in the LAD on LV function using 3D STE at rest. The study found that LS values negatively correlated with the severity of LAD systolic compression and significantly decreased in patients with isolated MB in the LAD when systolic compression was > 50%. However, LS was similar between patients with systolic compression < 50% and patients without MB. Previous studies evaluating strain imaging during DSE, have mainly included patients with systolic compression [26]. Systolic compression has been observed in 0.5–16% of patients undergoing ICA, whereas only 21–46% of intramyocardial coronary arteries on coronary CTA demonstrate systolic compression [20]. A previous study demonstrated that the depth of MB was associated with the likelihood of systolic compression [28]. However, we did not find differences in myocardial strain between patients with superficial or deep course of the MB segment. Notably, in patients with suspected obstructive CAD, guidelines recommend the use of invasive coronary angiography as the first diagnostic test only in selected patients with a high clinical likelihood or features of high event risk [29].

An additional finding in our study was that in patients with MB in the LAD, strain parameters including LS, LSr and RS correlated with stress myocardial blood flow measured by PET. This is in line with a recent study showing that 2D-strain values were significantly correlated with myocardial blood flow measured by PET during stress in patients with suspected CAD and normal LVEF [30] as well as previous results indicating that MB without systolic compression is not associated with reduced myocardial perfusion during vasodilator stress [11].

### Study limitations

A strength of our study is that all patients had detailed evaluation of coronary atherosclerosis and obstructive CAD by the combination of coronary CTA, PET perfusion imaging, ICA and invasive FFR measurements. This enabled ruling out effects of CAD on hemodynamic of MB and comparison of patients with MB and CAD.

Our study is limited by small number of eligible patients with MB in the original cohort of 50 patients. Furthermore, all systolic compressions were less than 50%. However, systolic compression is a dynamic phenomenon not always seen in a resting ICA [22]. Furthermore, systolic compression might worsen in some patients during exercise. Patients with delayed myocardial relaxation or upstream coronary stenoses might also be more susceptible to MB induced ischemia than our patients. Moreover, MB might lower the threshold for ischemic injury in type 2 myocardial ischemia. In this study, we did not evaluate the reproducibility of speckle tracking. However, we previously reported that analysis of 2D strain during DSE was feasible with low inter- and intra-observer variability [16], except PSI values that showed relatively high variation during peak stress. Finally, Longer-term outcome data were unavailable for this study, so we could not determine whether strain is useful in predicting cardiovascular outcomes in patients with an MB.

## Conclusions

Myocardial strain assessed by speckle tracking echocardiography during high dose dobutamine stress is not affected in patients with MB in the LAD without significant systolic compression.

## Data Availability

No datasets were generated or analysed during the current study.
